# *trans*-4,5-Dihydr­oxy-1,3-bis­(4-methoxy­phen­yl)imidazolidine-2-thione

**DOI:** 10.1107/S1600536809042779

**Published:** 2009-10-23

**Authors:** Zhenfeng Zhang, Jiange Wang, Guisheng Zhang

**Affiliations:** a College of Chemistry and Environmental Science, Henan Normal University, Xinxiang 453007, People’s Republic of China; b College of Chemistry, Luoyang Normal University, Xinxiang 453007, People’s Republic of China

## Abstract

In the title compound, C_17_H_18_N_2_O_4_S, where one of the *N-*4-methoxy­phenyl fragments is disordered over two sets of sites, the five-membered ring exhibits a nearly half-chair conformation and the two hydroxyl groups lie on opposite sides of the five-membered ring. In the crystal, the mol­ecules are linked into sheets parallel to (100) *via* O—H⋯O and O—H⋯S hydrogen bonds.

## Related literature

For the bioactivity of imidazolidine-2-one derivatives, see: Lam *et al.* (1994 ▶); Lenzen & Ahmad (2001 ▶); Perronnet & Teche (1973 ▶). For related structures, see: Zhang *et al.* (2007 ▶, 2009 ▶). For hydrogen-bond motifs, see: Bernstein *et al.* (1995 ▶). For puckering parameters, see: Cremer & Pople (1975 ▶).
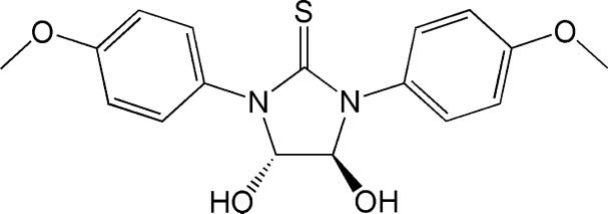


## Experimental

### 

#### Crystal data

C_17_H_18_N_2_O_4_S
*M* *_r_* = 346.39
Monoclinic, 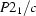

*a* = 13.9807 (12) Å
*b* = 12.1789 (11) Å
*c* = 10.0958 (9) Å
β = 93.815 (1)°
*V* = 1715.2 (3) Å^3^
*Z* = 4
Mo *K*α radiation
μ = 0.21 mm^−1^
*T* = 294 K
0.49 × 0.35 × 0.34 mm

#### Data collection

Bruker SMART CCD diffractometer
Absorption correction: multi-scan (*SADABS*; Sheldrick, 2003 ▶) *T*_min_ = 0.904, *T*_max_ = 0.931
12720 measured reflections
3179 independent reflections
2654 reflections with *I* > 2σ(*I*)
*R*_int_ = 0.019

#### Refinement

*R*[*F* ^2^ > 2σ(*F* ^2^)] = 0.041
*wR*(*F* ^2^) = 0.112
*S* = 1.03
3179 reflections
244 parameters
10 restraints
H-atom parameters constrained
Δρ_max_ = 0.41 e Å^−3^
Δρ_min_ = −0.60 e Å^−3^

### 

Data collection: *SMART* (Bruker, 1997 ▶); cell refinement: *SAINT* (Bruker, 1997 ▶); data reduction: *SAINT*; program(s) used to solve structure: *SHELXS97* (Sheldrick, 2008 ▶); program(s) used to refine structure: *SHELXL97* (Sheldrick, 2008 ▶); molecular graphics: *SHELXTL* (Sheldrick, 2008 ▶); software used to prepare material for publication: *SHELXTL*.

## Supplementary Material

Crystal structure: contains datablocks I. DOI: 10.1107/S1600536809042779/fl2268sup1.cif

Structure factors: contains datablocks I. DOI: 10.1107/S1600536809042779/fl2268Isup2.hkl

Additional supplementary materials:  crystallographic information; 3D view; checkCIF report

## Figures and Tables

**Table 1:** Hydrogen-bond geometry (Å, °)

*D*—H⋯*A*	*D*—H	H⋯*A*	*D*⋯*A*	*D*—H⋯*A*
O2—H2⋯O1^i^	0.82	1.99	2.7971 (19)	169
O1—H1⋯S1^ii^	0.82	2.40	3.1799 (14)	158

Symmetry codes: (i) 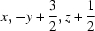; (ii) 
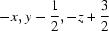
.

**Fig. 1.**
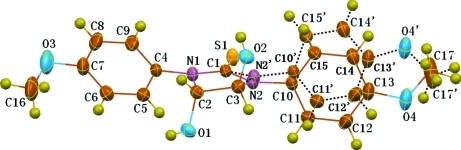
The molecular structure of (I), showing the atom-labelling scheme. Displacement ellipsoids are drawn at the 30% probability level and H atoms are shown as small spheres of arbitrary radii.

**Fig. 2.**
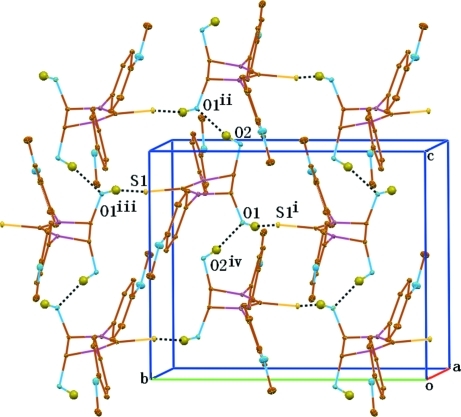
Part of the crystal structure of (I), showing the formation of a (100) sheet. For the sake of clarity, H atoms not involved in the motif shown have been omitted. Intermolecular interactions are represented by dashed lines. Selected atoms are labelled. [Symmetry codes: (i) -x,y - 1/2,-z + 3/2; (ii) x + 1,-y + 3/2,z + 1/2; (iii) -x,y + 1/2,-z + 3/2; (iv) x,-y + 3/2,z + 1/2].

## supplementary crystallographic information

### Comment

Imidazolidine-2-one derivates often exhibit powerful bioactivitiy, as herbicides (Perronnet & Teche,1973), antidiabetics (Lenzen & Ahmad, 2001) and anti-HIV agents (Lam *et al.*, 1994). Enders and his workers have earlier reported the synthesis and use of 4,5-dihydroxyimidazolidine-2-thiones. However, to our knowledge, there are few *N*,*N*'-diarylsubstituted 4,5-dihydroxyimidazolidine-2-thiones reported so far. As a continuation of our structural studies of such compounds (Zhang *et al.*, 2009), we report here the molecular and supramolecular structures of (I) (Fig. 1).

In (I), the N2-containing (4-methoxyphenyl)imino group is disordered over two sites with refined occupancies of 0.747 (2) and 0.253 (2) (Fig. 1). The five-membered ring adopts a nearly half-chair conformation; the total puckering amplitudes Q_2_ (Cremer & Pople, 1975) are 0.247 (5) and 0.187 (11) Å, and the ring puckering parameters φ_2_ are 302.1 (9) and 333 (5)°, respectively, for the atom sequences, N1—C1—N2—C3—C2 and N1—C1—N2'—C3—C2. For an idealized half-chair, the ring puckering angle is φ_2_ = (36k + 18) ° (where k = zero or an integer). Therefore, the conformation for the five-membered ring in (I) is markedly different from that found in our previously reported compound (Zhang *et al.*, 2007), where the five-membered ring shows a perfect envelope conformation. The difference in conformation is mainly attributed to van der Waals repulsions between the five-membered ring and its N1 and N2 phenyl substituents. Due to the existence of the van der Waals repulsions, the aryl groups exhibit nearly perpendicular orientations to the five-membered ring with the dihedral angle between the planes C4–C9 and C1/N1/C2 being 73.1 (2) °. Meanwhile, the dihedral angle between the planes C10–C15 and C1/N2/C3 is 89.7 (9) °. In addition, the C7 and C13 methoxy groups adopt closely coplanar orientations, respectively to their attached aryl rings, as shown by the torsion angles of C6—C7—O3—C16 [5.4 (4) °] and C14—C13—O4—C17 [6.4 (13) °]. Interestingly, the molecule (I) adopts a *trans* configuration (Fig. 1); the two hydroxyl groups lie on opposite sides of the five-membered ring. In view of the same *trans* configuration in 4,5-dihydroxyimidazolidine-2-thiones (Zhang *et al.*, 2007; Zhang *et al.*, 2009), we can draw a general conclusion that this *trans*-configuration is probably ubiquitous in 4,5-dihydroxyimidazolidine- 2-thiones.

The heterocyclic geometries of (I) also present some unexpected features. The C1—N1[1.366 (2) Å] and C1—N2 [1.352 (6) Å] bonds are significantly longer than the corresponding bonds in 4,5-dihydroxyimidazolidine-2-thione [1.335 (2) and 1.336 (2) Å; Zhang *et al.*, 2007]. Conversely, the C1-S1[1.669 (2) Å] and C2—C3 [1.523 (2) Å] bonds are shorter than the corresponding bonds in 4,5-dihydroxyimidazolidine-2-thione [1.684 (2) and 1.537 (2) Å, respectively].

In analyzing the supramolecular structure of (I), for the sake of simplicity, we shall omit any consideration of the intermolecular C—H···O interactions involving a C—H bond from an aromatic ring, which is far too long to be significant. Thus, molecules of (I) are linked into sheets by only two independent O—H···S and O—H···O hydrogen bonds (Table 1), the formation of which is readily analyzed in terms of two one-dimensional substructures. In the first substructure, hydroxyl atom O1 in the molecule at (*x*,*y*,*z*) acts as a hydrogen-bond donor to thiocarbonyl atom S1 in the molecule at (-*x*, *y* - 1/2,-*z* + 3/2), so forming a C_2_^2^(6) (Bernstein *et al.*,1995) chain along [010] and generated by 2_1_ screw axis along (0,*y*,3/4) (Fig. 2). Similarly in the second substructure, hydroxyl atom O2 in the molecule at (*x*,*y*,*z*) acts as a hydrogen-bond donor to hydroxyl atom O1 in the molecule at (*x*,-*y* + 3/2,*z* + 1/2), so forming a C(5) (Bernstein *et al.*, 1995) chain parallel to [001], this time generated by a 2_1_ screw along (1/8,3/4,*z*) (Fig. 2). The combination of the two chain motifs is sufficient to link all the molecules into a sheet parallel to (100). Two such sheets pass through each unit cell; in each sheet, there are both enantiomers of (I); there are no direction-specific interactions between adjacent sheets, in particular C—H···π hydrogen bonds and π-π stacking interactions are absent.

### Experimental

Into a three-necked round-bottomed flask equipped with a stirrer were introduced 1,3-bis(4-methoxyphenyl)thiourea(0.1 mol), glyoxal (40%, 18 g) and ethanol (95%, 30 ml). The mixture was then refluxed with stirring for *ca* 30 min and thereafter the solvent was removed; the residue was washed with cold ethanol and the resulting solid product was recrystallized from hot ethanol to give crystals of (I).

^1^H NMR(DMSO, 400 MHz) of (I): δ 7.36–6.95 (m, 8H), δ 7.1 (d, J = 8.0 Hz, 2H), δ 5.08 (d, J = 8.4 Hz, 2H), δ 3.76 (s, 6H).

### Refinement

The hydroxyl H atoms in (I) were found in a difference Fourier map and then freely refined. All other H atoms were positioned geometrically (aromatic C—H = 0.93 Å, methyl C—H = 0.96 Å, methyne C—H = 0.98 Å and O—H = 0.82 Å) and refined using a riding model [*U*_iso_(H) = 1.2*U*_eq_ (aromatic and methyne C) and 1.5 *U*_eq_(methyl C and hydroxy O)]. The N2-containing (4-methoxyphenyl)imino group was found to be disordered, and therefore was modelled over two sets of positions, with a refined major occupancy of 0.747 (2). 10 Geometric displacement-parameter restraints were applied to the disordered part. They are: *DFIX* 1.37 0.02 C10' C11'; *DFIX* 1.38 0.02 C11' C12'; *DFIX* 1.37 0.02 C10' C15' ; *DFIX* 1.37 0.02 C1 N2; *DFIX* 1.37 0.02 C1 N2'; *DFIX* 1.46 0.02 C3 N2; *DFIX* 1.46 0.02 C3 N2' ; *DFIX* 1.42 0.02 N2' C10'; *DFIX* 1.42 0.02 O4' C17'; *DFIX* 1.42 0.02 O4 C17

### Crystal data

**Table:**

C_17_H_18_N_2_O_4_S	*F*(000) = 728
*M**_r_* = 346.39	*D*_x_ = 1.341 Mg m^−^^3^
Monoclinic, *P*2_1_/*c*	Mo *K*α radiation, λ = 0.71073 Å
*a* = 13.9807 (12) Å	Cell parameters from 4930 reflections
*b* = 12.1789 (11) Å	θ = 2.6–26.9°
*c* = 10.0958 (9) Å	µ = 0.21 mm^−^^1^
β = 93.815 (1)°	*T* = 294 K
*V* = 1715.2 (3) Å^3^	Block, colorless
*Z* = 4	0.49 × 0.35 × 0.34 mm

### Data collection

**Table:**

Bruker SMART CCD diffractometer	3179 independent reflections
Radiation source: fine-focus sealed tube	2654 reflections with *I* > 2σ(*I*)
graphite	*R*_int_ = 0.019
phi and ω scans	θ_max_ = 25.5°, θ_min_ = 2.6°
Absorption correction: multi-scan (*SADABS*; Sheldrick, 2003)	*h* = −16→16
*T*_min_ = 0.904, *T*_max_ = 0.931	*k* = −14→14
12720 measured reflections	*l* = −12→11

### Refinement

**Table:**

Refinement on *F*^2^	Primary atom site location: structure-invariant direct methods
Least-squares matrix: full	Secondary atom site location: difference Fourier map
*R*[*F*^2^ > 2σ(*F*^2^)] = 0.041	Hydrogen site location: inferred from neighbouring sites
*wR*(*F*^2^) = 0.112	H-atom parameters constrained
*S* = 1.03	*w* = 1/[σ^2^(*F*_o_^2^) + (0.0522*P*)^2^ + 0.751*P*] where *P* = (*F*_o_^2^ + 2*F*_c_^2^)/3
3179 reflections	(Δ/σ)_max_ = 0.001
244 parameters	Δρ_max_ = 0.41 e Å^−^^3^
10 restraints	Δρ_min_ = −0.60 e Å^−^^3^

### Special details

**Table:**

Geometry. All e.s.d.'s (except the e.s.d. in the dihedral angle between two l.s. planes) are estimated using the full covariance matrix. The cell e.s.d.'s are taken into account individually in the estimation of e.s.d.'s in distances, angles and torsion angles; correlations between e.s.d.'s in cell parameters are only used when they are defined by crystal symmetry. An approximate (isotropic) treatment of cell e.s.d.'s is used for estimating e.s.d.'s involving l.s. planes.
Refinement. Refinement of *F*^2^ against ALL reflections. The weighted *R*-factor *wR* and goodness of fit *S* are based on *F*^2^, conventional *R*-factors *R* are based on *F*, with *F* set to zero for negative *F*^2^. The threshold expression of *F*^2^ > σ(*F*^2^) is used only for calculating *R*-factors(gt) *etc*. and is not relevant to the choice of reflections for refinement. *R*-factors based on *F*^2^ are statistically about twice as large as those based on *F*, and *R*- factors based on ALL data will be even larger.

### Fractional atomic coordinates and isotropic or equivalent isotropic displacement parameters (Å^2^)

**Table:**

	*x*	*y*	*z*	*U*_iso_*/*U*_eq_	Occ. (<1)
N2	0.1894 (5)	0.8182 (5)	0.8627 (11)	0.0376 (14)	0.746 (2)
C10	0.2878 (3)	0.8426 (4)	0.8958 (3)	0.0387 (8)	0.746 (2)
C11	0.3522 (2)	0.8265 (2)	0.7987 (3)	0.0538 (7)	0.746 (2)
H11	0.3295	0.8116	0.7119	0.065*	0.746 (2)
C12	0.4492 (2)	0.8325 (3)	0.8299 (3)	0.0644 (8)	0.746 (2)
H12	0.4920	0.8229	0.7640	0.077*	0.746 (2)
C13	0.4833 (2)	0.8528 (2)	0.9598 (3)	0.0532 (7)	0.746 (2)
C14	0.4205 (2)	0.8754 (3)	1.0555 (3)	0.0542 (8)	0.746 (2)
H14	0.4432	0.8936	1.1413	0.065*	0.746 (2)
C15	0.3222 (2)	0.8705 (2)	1.0222 (3)	0.0480 (7)	0.746 (2)
H15	0.2793	0.8863	1.0862	0.058*	0.746 (2)
C17	0.6212 (9)	0.861 (2)	1.1138 (17)	0.0922 (15)	0.746 (2)
H17A	0.5976	0.9264	1.1523	0.138*	0.746 (2)
H17B	0.6898	0.8640	1.1139	0.138*	0.746 (2)
H17C	0.6031	0.7982	1.1648	0.138*	0.746 (2)
O4	0.58130 (14)	0.8499 (2)	0.9808 (3)	0.0785 (7)	0.746 (2)
N2'	0.1897 (16)	0.8218 (15)	0.883 (4)	0.0376 (14)	0.254 (2)
C10'	0.2843 (11)	0.8530 (16)	0.9341 (14)	0.0387 (8)	0.254 (2)
C11'	0.3624 (6)	0.8085 (8)	0.8804 (9)	0.0538 (7)	0.254 (2)
H11'	0.3560	0.7707	0.8003	0.065*	0.254 (2)
C12'	0.4518 (7)	0.8206 (10)	0.9478 (12)	0.0644 (8)	0.254 (2)
H12'	0.5064	0.7924	0.9123	0.077*	0.254 (2)
C13'	0.4586 (7)	0.8747 (9)	1.0675 (13)	0.0532 (7)	0.254 (2)
C14'	0.3778 (6)	0.9179 (8)	1.1206 (9)	0.0542 (8)	0.254 (2)
H14'	0.3837	0.9566	1.2001	0.065*	0.254 (2)
C15'	0.2897 (7)	0.9037 (8)	1.0563 (9)	0.0480 (7)	0.254 (2)
H15'	0.2345	0.9277	1.0941	0.058*	0.254 (2)
C17'	0.629 (3)	0.854 (7)	1.100 (6)	0.0922 (15)	0.254 (2)
H17D	0.6417	0.7791	1.1256	0.138*	0.254 (2)
H17E	0.6805	0.8998	1.1347	0.138*	0.254 (2)
H17F	0.6243	0.8588	1.0044	0.138*	0.254 (2)
O4'	0.5427 (4)	0.8885 (6)	1.1491 (8)	0.0785 (7)	0.254 (2)
S1	0.12664 (3)	1.02415 (4)	0.81566 (5)	0.04968 (17)	
O1	0.06318 (10)	0.66792 (11)	0.68364 (12)	0.0507 (4)	
H1	0.0227	0.6199	0.6696	0.076*	
O2	0.15350 (10)	0.68187 (11)	1.02357 (12)	0.0498 (3)	
H2	0.1261	0.7311	1.0616	0.075*	
O3	−0.33055 (13)	0.9381 (2)	0.6034 (2)	0.1059 (6)	
N1	0.03525 (10)	0.82649 (12)	0.81002 (15)	0.0404 (3)	
C1	0.11643 (11)	0.88826 (14)	0.83077 (16)	0.0368 (4)	
C2	0.05733 (13)	0.70931 (15)	0.81329 (17)	0.0409 (4)	
H2A	0.0101	0.6685	0.8611	0.049*	
C3	0.15534 (13)	0.70725 (14)	0.88879 (17)	0.0405 (4)	
H3	0.1963	0.6541	0.8466	0.049*	
C4	−0.05667 (12)	0.86336 (15)	0.75615 (17)	0.0402 (4)	
C5	−0.06968 (13)	0.90132 (16)	0.62842 (18)	0.0446 (4)	
H5	−0.0168	0.9098	0.5782	0.054*	
C6	−0.16006 (14)	0.92724 (17)	0.5726 (2)	0.0509 (5)	
H6	−0.1680	0.9526	0.4857	0.061*	
C7	−0.23823 (14)	0.9148 (2)	0.6480 (2)	0.0602 (6)	
C8	−0.22540 (15)	0.8775 (2)	0.7771 (2)	0.0737 (7)	
H8	−0.2782	0.8698	0.8277	0.088*	
C9	−0.13567 (14)	0.8517 (2)	0.8319 (2)	0.0601 (6)	
H9	−0.1277	0.8267	0.9190	0.072*	
C16	−0.34805 (19)	0.9682 (3)	0.4689 (3)	0.1059 (6)	
H16A	−0.3167	1.0367	0.4531	0.159*	
H16B	−0.4158	0.9761	0.4489	0.159*	
H16C	−0.3235	0.9124	0.4132	0.159*	

### Atomic displacement parameters (Å^2^)

**Table:**

	*U*^11^	*U*^22^	*U*^33^	*U*^12^	*U*^13^	*U*^23^
N2	0.0346 (8)	0.0367 (9)	0.041 (4)	−0.0025 (6)	−0.0037 (13)	0.0023 (13)
C10	0.0363 (11)	0.0352 (15)	0.044 (3)	0.0000 (9)	−0.0051 (17)	−0.0031 (18)
C11	0.0475 (14)	0.0643 (16)	0.0496 (16)	−0.0027 (12)	0.0039 (14)	−0.0145 (15)
C12	0.0405 (14)	0.076 (2)	0.0784 (19)	−0.0006 (13)	0.0130 (14)	−0.0227 (17)
C13	0.0337 (15)	0.0461 (15)	0.0786 (19)	−0.0015 (11)	−0.0059 (14)	0.0030 (14)
C14	0.0476 (19)	0.0632 (18)	0.0502 (17)	−0.0093 (16)	−0.0092 (14)	0.0047 (13)
C15	0.0443 (17)	0.0579 (19)	0.0419 (16)	−0.0056 (13)	0.0029 (12)	−0.0013 (12)
C17	0.052 (3)	0.098 (4)	0.122 (5)	−0.017 (2)	−0.030 (2)	0.028 (3)
O4	0.0357 (10)	0.0820 (15)	0.1161 (19)	−0.0034 (10)	−0.0094 (11)	0.0010 (13)
N2'	0.0346 (8)	0.0367 (9)	0.041 (4)	−0.0025 (6)	−0.0037 (13)	0.0023 (13)
C10'	0.0363 (11)	0.0352 (15)	0.044 (3)	0.0000 (9)	−0.0051 (17)	−0.0031 (18)
C11'	0.0475 (14)	0.0643 (16)	0.0496 (16)	−0.0027 (12)	0.0039 (14)	−0.0145 (15)
C12'	0.0405 (14)	0.076 (2)	0.0784 (19)	−0.0006 (13)	0.0130 (14)	−0.0227 (17)
C13'	0.0337 (15)	0.0461 (15)	0.0786 (19)	−0.0015 (11)	−0.0059 (14)	0.0030 (14)
C14'	0.0476 (19)	0.0632 (18)	0.0502 (17)	−0.0093 (16)	−0.0092 (14)	0.0047 (13)
C15'	0.0443 (17)	0.0579 (19)	0.0419 (16)	−0.0056 (13)	0.0029 (12)	−0.0013 (12)
C17'	0.052 (3)	0.098 (4)	0.122 (5)	−0.017 (2)	−0.030 (2)	0.028 (3)
O4'	0.0357 (10)	0.0820 (15)	0.1161 (19)	−0.0034 (10)	−0.0094 (11)	0.0010 (13)
S1	0.0414 (3)	0.0359 (3)	0.0700 (4)	−0.00154 (19)	−0.0095 (2)	−0.0005 (2)
O1	0.0596 (9)	0.0473 (8)	0.0449 (7)	−0.0153 (6)	0.0015 (6)	−0.0073 (6)
O2	0.0591 (9)	0.0482 (8)	0.0417 (7)	0.0025 (6)	0.0011 (6)	0.0083 (6)
O3	0.0481 (8)	0.1623 (18)	0.1038 (13)	0.0091 (9)	−0.0214 (8)	0.0229 (12)
N1	0.0348 (8)	0.0394 (8)	0.0463 (8)	−0.0035 (6)	−0.0028 (6)	−0.0003 (6)
C1	0.0329 (9)	0.0419 (9)	0.0353 (8)	−0.0004 (7)	−0.0013 (7)	−0.0010 (7)
C2	0.0426 (10)	0.0399 (10)	0.0402 (9)	−0.0087 (8)	0.0037 (7)	0.0012 (7)
C3	0.0429 (10)	0.0372 (9)	0.0412 (9)	−0.0008 (7)	0.0026 (7)	0.0024 (7)
C4	0.0327 (9)	0.0442 (10)	0.0433 (10)	−0.0041 (7)	−0.0007 (7)	−0.0047 (8)
C5	0.0412 (10)	0.0480 (10)	0.0449 (10)	−0.0015 (8)	0.0044 (8)	−0.0018 (8)
C6	0.0511 (11)	0.0536 (12)	0.0467 (10)	0.0004 (9)	−0.0072 (9)	0.0009 (9)
C7	0.0379 (11)	0.0709 (14)	0.0702 (14)	−0.0008 (10)	−0.0092 (10)	−0.0009 (11)
C8	0.0370 (11)	0.117 (2)	0.0681 (15)	−0.0030 (12)	0.0095 (10)	0.0065 (14)
C9	0.0434 (11)	0.0916 (17)	0.0452 (11)	−0.0045 (11)	0.0029 (9)	0.0067 (11)
C16	0.0481 (8)	0.1623 (18)	0.1038 (13)	0.0091 (9)	−0.0214 (8)	0.0229 (12)

### Geometric parameters (Å, °)

**Table:**

N2—C1	1.352 (6)	C15'—H15'	0.9300
N2—C10	1.426 (6)	C17'—O4'	1.40 (2)
N2—C3	1.462 (6)	C17'—H17D	0.9600
C10—C15	1.376 (4)	C17'—H17E	0.9600
C10—C11	1.388 (5)	C17'—H17F	0.9600
C11—C12	1.374 (4)	S1—C1	1.669 (2)
C11—H11	0.9300	O1—C2	1.410 (2)
C12—C13	1.388 (5)	O1—H1	0.8200
C12—H12	0.9300	O2—C3	1.397 (2)
C13—O4	1.372 (3)	O2—H2	0.8200
C13—C14	1.376 (5)	O3—C7	1.368 (3)
C14—C15	1.394 (4)	O3—C16	1.413 (4)
C14—H14	0.9300	N1—C1	1.366 (2)
C15—H15	0.9300	N1—C4	1.434 (2)
C17—O4	1.426 (17)	N1—C2	1.460 (2)
C17—H17A	0.9600	C2—C3	1.523 (2)
C17—H17B	0.9600	C2—H2A	0.9800
C17—H17C	0.9600	C3—H3	0.9800
N2'—C1	1.382 (17)	C4—C5	1.371 (3)
N2'—C10'	1.438 (17)	C4—C9	1.392 (3)
N2'—C3	1.478 (17)	C5—C6	1.385 (3)
C10'—C11'	1.364 (14)	C5—H5	0.9300
C10'—C15'	1.377 (13)	C6—C7	1.381 (3)
C11'—C12'	1.391 (12)	C6—H6	0.9300
C11'—H11'	0.9300	C7—C8	1.381 (3)
C12'—C13'	1.374 (18)	C8—C9	1.373 (3)
C12'—H12'	0.9300	C8—H8	0.9300
C13'—C14'	1.385 (15)	C9—H9	0.9300
C13'—O4'	1.400 (12)	C16—H16A	0.9600
C14'—C15'	1.365 (12)	C16—H16B	0.9600
C14'—H14'	0.9300	C16—H16C	0.9600
			
C1—N2—C10	128.7 (5)	C3—O2—H2	109.5
C1—N2—C3	112.1 (5)	C7—O3—C16	118.0 (2)
C10—N2—C3	118.1 (4)	C1—N1—C4	126.88 (15)
C15—C10—C11	119.1 (4)	C1—N1—C2	111.25 (14)
C15—C10—N2	122.8 (6)	C4—N1—C2	119.84 (14)
C11—C10—N2	117.8 (5)	N2—C1—N1	107.1 (3)
C12—C11—C10	120.5 (3)	N2—C1—N2'	9(2)
C12—C11—H11	119.7	N1—C1—N2'	108.9 (8)
C10—C11—H11	119.7	N2—C1—S1	125.4 (3)
C11—C12—C13	119.8 (3)	N1—C1—S1	127.39 (13)
C11—C12—H12	120.1	N2'—C1—S1	123.4 (7)
C13—C12—H12	120.1	O1—C2—N1	110.71 (14)
O4—C13—C14	125.1 (3)	O1—C2—C3	110.66 (15)
O4—C13—C12	114.7 (3)	N1—C2—C3	102.08 (13)
C14—C13—C12	120.2 (3)	O1—C2—H2A	111.0
C13—C14—C15	119.2 (3)	N1—C2—H2A	111.0
C13—C14—H14	120.4	C3—C2—H2A	111.0
C15—C14—H14	120.4	O2—C3—N2	114.0 (5)
C10—C15—C14	120.8 (3)	O2—C3—N2'	106.0 (16)
C10—C15—H15	119.6	N2—C3—N2'	8(2)
C14—C15—H15	119.6	O2—C3—C2	114.63 (15)
C13—O4—C17	117.8 (5)	N2—C3—C2	100.8 (3)
C1—N2'—C10'	128.5 (15)	N2'—C3—C2	104.4 (7)
C1—N2'—C3	109.5 (12)	O2—C3—H3	109.0
C10'—N2'—C3	121.9 (14)	N2—C3—H3	109.0
C11'—C10'—C15'	122.4 (13)	N2'—C3—H3	113.9
C11'—C10'—N2'	119.6 (18)	C2—C3—H3	109.0
C15'—C10'—N2'	116 (2)	C5—C4—C9	119.39 (17)
C10'—C11'—C12'	118.8 (10)	C5—C4—N1	121.47 (16)
C10'—C11'—H11'	120.6	C9—C4—N1	118.93 (17)
C12'—C11'—H11'	120.6	C4—C5—C6	121.34 (18)
C13'—C12'—C11'	119.2 (9)	C4—C5—H5	119.3
C13'—C12'—H12'	120.4	C6—C5—H5	119.3
C11'—C12'—H12'	120.4	C7—C6—C5	118.96 (19)
C12'—C13'—C14'	120.8 (9)	C7—C6—H6	120.5
C12'—C13'—O4'	125.5 (10)	C5—C6—H6	120.5
C14'—C13'—O4'	113.6 (10)	O3—C7—C8	116.0 (2)
C15'—C14'—C13'	120.1 (9)	O3—C7—C6	124.0 (2)
C15'—C14'—H14'	120.0	C8—C7—C6	119.92 (19)
C13'—C14'—H14'	120.0	C9—C8—C7	120.9 (2)
C14'—C15'—C10'	118.5 (11)	C9—C8—H8	119.6
C14'—C15'—H15'	120.8	C7—C8—H8	119.6
C10'—C15'—H15'	120.8	C8—C9—C4	119.5 (2)
O4'—C17'—H17D	109.5	C8—C9—H9	120.2
O4'—C17'—H17E	109.5	C4—C9—H9	120.2
H17D—C17'—H17E	109.5	O3—C16—H16A	109.5
O4'—C17'—H17F	109.5	O3—C16—H16B	109.5
H17D—C17'—H17F	109.5	H16A—C16—H16B	109.5
H17E—C17'—H17F	109.5	O3—C16—H16C	109.5
C13'—O4'—C17'	118 (2)	H16A—C16—H16C	109.5
C2—O1—H1	109.5	H16B—C16—H16C	109.5
			
C1—N2—C10—C15	84.6 (12)	C10'—N2'—C1—N2	108 (10)
C3—N2—C10—C15	−82.6 (9)	C3—N2'—C1—N2	−76 (6)
C1—N2—C10—C11	−102.0 (11)	C10'—N2'—C1—N1	−172 (3)
C3—N2—C10—C11	90.9 (9)	C3—N2'—C1—N1	4(3)
C15—C10—C11—C12	3.5 (7)	C10'—N2'—C1—S1	1(5)
N2—C10—C11—C12	−170.2 (4)	C3—N2'—C1—S1	176.9 (12)
C10—C11—C12—C13	1.1 (5)	C1—N1—C2—O1	−97.38 (16)
C11—C12—C13—O4	176.0 (3)	C4—N1—C2—O1	67.5 (2)
C11—C12—C13—C14	−5.0 (5)	C1—N1—C2—C3	20.43 (18)
O4—C13—C14—C15	−176.9 (3)	C4—N1—C2—C3	−174.67 (14)
C12—C13—C14—C15	4.1 (5)	C1—N2—C3—O2	−100.6 (7)
C11—C10—C15—C14	−4.4 (7)	C10—N2—C3—O2	68.6 (9)
N2—C10—C15—C14	169.0 (4)	C1—N2—C3—N2'	−95 (8)
C13—C14—C15—C10	0.6 (5)	C10—N2—C3—N2'	75 (7)
C14—C13—O4—C17	6.4 (13)	C1—N2—C3—C2	22.7 (8)
C12—C13—O4—C17	−174.6 (13)	C10—N2—C3—C2	−168.1 (7)
C1—N2'—C10'—C11'	−123 (3)	C1—N2'—C3—O2	−113 (2)
C3—N2'—C10'—C11'	62 (4)	C10'—N2'—C3—O2	64 (3)
C1—N2'—C10'—C15'	74 (4)	C1—N2'—C3—N2	73 (6)
C3—N2'—C10'—C15'	−102 (3)	C10'—N2'—C3—N2	−111 (10)
C15'—C10'—C11'—C12'	−4(2)	C1—N2'—C3—C2	9(3)
N2'—C10'—C11'—C12'	−166.4 (17)	C10'—N2'—C3—C2	−175 (3)
C10'—C11'—C12'—C13'	1.4 (19)	O1—C2—C3—O2	−143.51 (15)
C11'—C12'—C13'—C14'	−0.6 (18)	N1—C2—C3—O2	98.64 (16)
C11'—C12'—C13'—O4'	176.8 (10)	O1—C2—C3—N2	93.6 (5)
C12'—C13'—C14'—C15'	2.3 (16)	N1—C2—C3—N2	−24.3 (5)
O4'—C13'—C14'—C15'	−175.4 (8)	O1—C2—C3—N2'	101.0 (17)
C13'—C14'—C15'—C10'	−4.6 (17)	N1—C2—C3—N2'	−16.9 (17)
C11'—C10'—C15'—C14'	6(2)	C1—N1—C4—C5	64.1 (2)
N2'—C10'—C15'—C14'	168.7 (14)	C2—N1—C4—C5	−98.2 (2)
C12'—C13'—O4'—C17'	7(4)	C1—N1—C4—C9	−121.2 (2)
C14'—C13'—O4'—C17'	−176 (4)	C2—N1—C4—C9	76.4 (2)
C10—N2—C1—N1	−178.6 (9)	C9—C4—C5—C6	−0.7 (3)
C3—N2—C1—N1	−10.9 (9)	N1—C4—C5—C6	173.91 (17)
C10—N2—C1—N2'	−76 (7)	C4—C5—C6—C7	0.3 (3)
C3—N2—C1—N2'	92 (8)	C16—O3—C7—C8	−175.2 (3)
C10—N2—C1—S1	3.1 (14)	C16—O3—C7—C6	5.4 (4)
C3—N2—C1—S1	170.9 (4)	C5—C6—C7—O3	179.8 (2)
C4—N1—C1—N2	−170.5 (6)	C5—C6—C7—C8	0.4 (3)
C2—N1—C1—N2	−6.9 (6)	O3—C7—C8—C9	−180.0 (3)
C4—N1—C1—N2'	−179.4 (19)	C6—C7—C8—C9	−0.5 (4)
C2—N1—C1—N2'	−15.8 (19)	C7—C8—C9—C4	0.0 (4)
C4—N1—C1—S1	7.7 (3)	C5—C4—C9—C8	0.6 (3)
C2—N1—C1—S1	171.30 (13)	N1—C4—C9—C8	−174.2 (2)

### Hydrogen-bond geometry (Å, °)

**Table:**

*D*—H···*A*	*D*—H	H···*A*	*D*···*A*	*D*—H···*A*
O2—H2···O1^i^	0.82	1.99	2.7971 (19)	169
O1—H1···S1^ii^	0.82	2.40	3.1799 (14)	158

Symmetry codes: (i) *x*, −*y*+3/2, *z*+1/2; (ii) −*x*, *y*−1/2, −*z*+3/2.
